# COVID-19 vaccine intercountry distribution inequality and its underlying factors: a combined concentration index analysis and multiple linear regression analysis

**DOI:** 10.3389/fpubh.2024.1348088

**Published:** 2024-03-21

**Authors:** Wafa Abu El Kheir-Mataria, Zeinab Khadr, Hassan El Fawal, Sungsoo Chun

**Affiliations:** ^1^Institute of Global Health and Human Ecology, The American University in Cairo, New Cairo, Egypt; ^2^The Social Research Center, The American University in Cairo, New Cairo, Egypt; ^3^Department of Statistics, Faculty of Economics and Political Sciences, Cairo University, Giza, Egypt

**Keywords:** COVID-19, vaccine distribution, equity, global health governance, concentration index, universal health coverage, health system strength, political power and economic power

## Abstract

**Introduction:**

Inequitable access to COVID-19 vaccines among countries is a pressing global health issue. Factors such as economic power, political power, political stability, and health system strength contribute to disparities in vaccine distribution. This study aims to assess the inequality in vaccine distribution among countries based on these factors and identify their relationship with COVID-19 vaccine distribution.

**Methods:**

A Concentration Index (CI) analysis was conducted to evaluate inequalities in the distribution of COVID-19 vaccines among countries based on four separate variables: GDP *per capita*, political stability (PS), World Power Index (WPI), and Universal Health Coverage (UHC). Additionally, Multiple Linear Regression (MLR) analysis was employed to explore the relationship between vaccine distribution and these independent variables. Two vaccine distribution variables were utilized for result reliability.

**Results:**

The analysis revealed significant inequalities in COVID-19 vaccine distribution according to the countries’ GDP/capita, PS, WPI, and UHC. However, the multiple linear regression analysis showed that there is no significant relationship between COVID-19 vaccine distribution and the countries’ GDP/capita and that UHC is the most influential factor impacting COVID-19 vaccine distribution and accessibility.

**Discussion:**

The findings underscore the complex interplay between economic, political, and health system factors in shaping vaccine distribution patterns. To improve the accessibility to vaccines in future pandemics, Global Health Governance (GHG) and countries should consider working on three areas; enhance political stabilities in countries, separate the political power from decision-making at the global level and most importantly support countries to achieve UHC.

## 1 Introduction

During the COVID-19 pandemic, health equity proves to be an issue of concern at both the national and international levels. At the national level, researchers have been concerned with disparities in the level of infection, consequences, and vaccination among different social groups within their countries (1–14). One of the major health equity concerns at the global level was the inequitable access to the COVID-19 vaccine, especially in the period following the start of COVID-19 vaccine production and before it became abundant. Global health governance (GHG) is responsible for the coordination of COVID-19 vaccine distribution equitably; however, this was not the case (15, 16). According to the Our World in Data website on 7 April 2022, the share of fully vaccinated people in high-income countries (HICs) and upper-middle-income countries (UMICs) reached 74.1% and 76.68%, respectively. In contrast, the share of fully vaccinated people in lower-middle-income countries (LMICs) and low-income countries (LICs) reached 50.51% and 11.51%, respectively. As for the share of partially vaccinated people, it was estimated to be 5.05%, 4.77%, 9.17%, and 3.26% in HICs, UMICs, LMICs, and LICs, respectively (17). These numbers indicate the presence of discrepancies in securing the vaccine across these country groups.

Several published studies discuss the reasons underlying inequities in COVID-19 vaccine distribution. Some studies discussed the influence of economic power in securing the vaccine as well as in controlling its distribution. Countries with high economic power can produce, trade, and control the value chain of COVID-19 vaccines (18). Their financing capacity enables the production as well as the purchase of needed vaccines. Countries’ purchasing power appeared to be determinative in the accessibility to the vaccine (19). Many HICs rushed to purchase the COVID-19 vaccine for their population even before its final approval (20). This action has affected the availability of the vaccine in other countries.

Other studies raised the issue of political power imbalance among actors and its role in vaccine accessibility. Political power has a dual role in COVID-19 vaccine accessibility, and it acts as a determinant as well as a consequence. Power has been detrimental to COVID-19 vaccine accessibility (21). Powerful countries compete to settle their ideological perspectives in the global health arena and use their power to influence less powerful countries’ accessibility to vaccines (22). Countries’ responses to the pandemic were influenced by their power resulting in vaccine nationalism (23). Western countries intentionally hoarded the vaccine for themselves and their allies ignoring other countries’ needs (24). On the other hand, countries aimed to gain power through higher accessibility to the vaccine. Fiddler stated that COVID-19-related decisions are influenced by geopolitical calculations. COVID-19 vaccine access is considered a source of political power (25). Countries with higher vaccine coverage are expected to have better chances for rapid economic recovery (26, 27). Furthermore, countries’ ability to manufacture the vaccine and decide on its distribution projects soft power and demonstrates ambition for geopolitical opportunities (28, 29).

On another front, several published studies discuss the reasons underlying inequalities in COVID-19 vaccine distribution related to countries’ health systems. These studies highlighted the limited countries’ capacities, poor infrastructure, inadequate supply chain capability, and limited technical expertise as main barriers to the manufacturing, storage, and administration of the COVID-19 vaccine in these countries, which in turn limited their access to the vaccine doses (30–34).

This research addresses two existing gaps in the literature. First, it provides quantitative measuring of the inequality in COVID-19 vaccine distribution by economic status, political stability (PS), political power, and health system strength. Second, it sheds light on the interplay between these factors with the aim of providing valuable insight into the root causes of these inequalities. Thus, the first objective of this study is to estimate the inequality in the distribution of COVID-19 vaccines among countries by their economic status, PS, political power, and health system strength. The second objective is to determine which of these factors are significantly related to the distribution of COVID-19 vaccines among countries.

## 2 Materials and methods

Given the objectives of the study, two types of analysis were used: concentration index (CI) analysis and multiple linear regression (MLR) analysis. CI analysis is used to determine inequalities in the distribution of the COVID-19 vaccines among countries by four separate variables. It is a recognized technique for evaluating disparities in the distribution of health-related outcomes. It is consistent with the study’s primary goal of evaluating the disparity in vaccine distribution among nations. Using CI analysis for four separate variables (political power, PS, economic status, and the strength of the health system) breaks down the analysis by these factors and helps identify specific dimensions of inequality, providing valuable insights into these factors’ contribution to the inequitable vaccine distribution.

MLR is a known method used to examine the relationship between a dependent variable and multiple independent variables. It allows this while controlling for other independent variables, enabling the identification of the significant factors influencing vaccine distribution. In this study, MLR helps in achieving the second objective by identifying the key drivers of vaccine distribution disparities among countries.

Together, CI analysis and MLR provide a comprehensive understanding of the inequities in COVID-19 vaccine distribution among countries and the underlying factors relating to this distribution.

### 2.1 Concentration index analysis

Different measures are used to assess inequality in health (35, 36). The most commonly used are the CI (37, 38), the Gini coefficient (39, 40), the index of dissimilarity (41), the range (42) and the slope and relative indices of inequality (37). The current research uses the CI approach to examine inequalities in COVID-19 vaccine distribution among countries using STATA 15.

**Figure 1:**
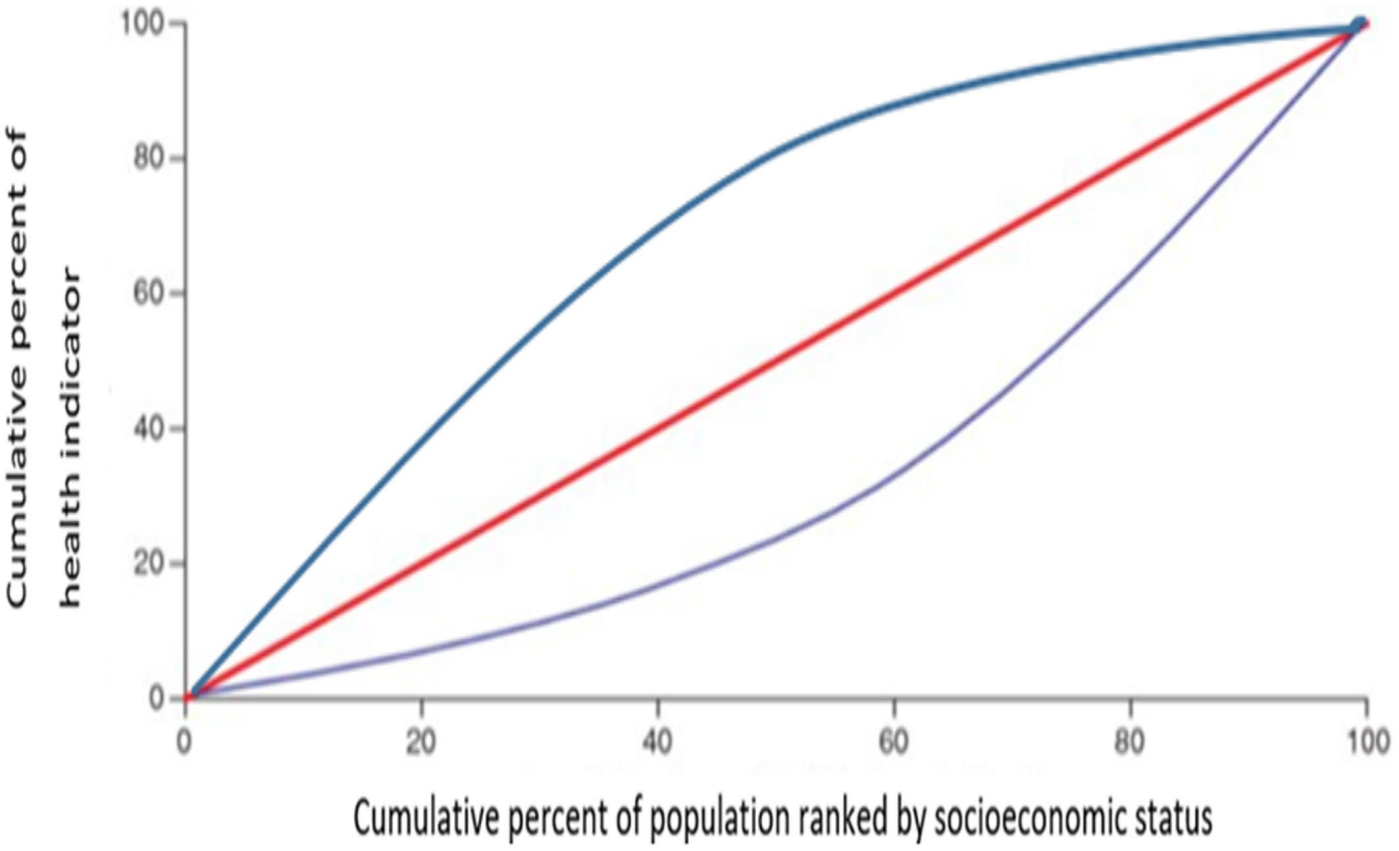
Concentration curve.

CI is an index used to measure inequalities in health that are associated systemically with socioeconomic status. It is calculated from the concentration curve. The concentration curve is plotted as follows: On the X-axis is the cumulative proportion of countries ranked according to their socioeconomic status (i.e., GDP/capita, for this study), starting from the least advantaged to the most advantaged. On the Y-axis is the cumulative proportion of health (i.e., vaccine distribution among countries, for this study). The CI is twice the area between the concentration curve and the diagonal (line of equality; Figure 1). Inequality is observed when the concentration curve is not aligned with the line of equality. The more convex the concentration curve, the higher the inequality (37, 43). The CI is calculated as follows:


$$CI=\frac{2}{N\mu }\overset{n}{\underset{i=1}{\sum }}h_{i}r_{i}-1-\frac{1}{N}$$

*N* = the number of categories of the social grouping

*h_i_* = the health variable (vaccine distribution)
*μ* = the mean of the health variable
*r_i_* = is the fractional rank of country i in the social grouping, with i = 1 for the lowest social grouping and i = N for the highest

### 2.2 Multiple linear regression analysis

MLR is a statistical technique that estimates the relationship between one dependent variable and two or more independent variables and how the dependent variable will change when independent variables change (44).

Assuming that the used health indicator (y), which in this case is COVID-19 vaccine distribution (i.e., TVH and VPP), is related to a number of factors, the relationship between the health indicator and these factors can be written using a linear regression model:


$$y=\beta_{0}+\beta_{1}X_{1}+\ldots +\beta_{n}X_{n}+\in$$

y = the predicted value of the dependent variable

$\beta_{0}$ = y-intercept, value of y when all other parameters are set to 0
$\beta_{1}X_{1}$ = the regression coefficient ($\beta_{1}$ ) of the first independent variable ($X_{1}$)
$\beta_{n}X_{n}$ = the regression coefficient of the last independent variable
ϵ = model error

MLR has three assumptions: there is no multicollinearity, which means that the independent variables are not highly correlated with each other. Homoscedasticity requires that the variance of the errors should be constant across all levels of the independent variables. Furthermore, autocorrelation requires that the residuals (errors) should be independent of each other.

To ensure the reliability of the MLR analysis results of this study, three tests were performed: a variance inflation factor (VIF) analysis was conducted to examine multicollinearity among independent variables. The results showed that all VIP scores were less than 5, meaning that multicollinearity is very law (45). A Breusch–Pagan test was performed to measure homoscedasticity. The result shows a high *p*-value (*p* = 0.980) suggesting that the all-independent data ensure homoscedasticity (46). The Durbin–Watson test was performed to detect autocorrelation in the residuals. The value of the Durbin–Watson test was 2.042, indicating no significant autocorrelation (47). The three aforementioned analyses along with the MLR, were performed using SPSS 26.

### 2.3 Data sources and study variables

For the COVID-19 vaccine distribution, two variables were used: first, total vaccinations per 100 people within a given population (TVH). TVH is the number of vaccine doses administered per 100 people within a given population, including booster doses. All doses are counted individually (17). Data for this indicator were extracted on 17 May 2022. Second, vaccine courses delivered as a proportion of the country’s population (VPP). VPP is the number of vaccine doses—in a full course for a given vaccine—as a proportion of the country’s population (48). Data for this indicator were extracted on 25 February 2022. These two indicators were chosen due to the credibility of their sources (the World in Data website and the UNICEF COVID-19 Vaccine Market Dashboard). The two different COVID-19 vaccine distribution indicators (TVH and VPP) were used separately as indicators for vaccine distribution among countries. This was performed to ascertain the credibility of the results, regardless of the indicator used.

Apart from VPP and TVH, four other variables were used in this study. These four variables served as stratifiers in the CI analysis and as independent variables in the MLR analysis. The first variable is GDP/capita (49). The second variable is the World Power Index (WPI), which is a composite index. It is composed of three subindexes that reflect the multi-dimensionality of the concept of state power. The three subindexes are the material capacities index (MCI), semi-material capacities index (SMCI), and immaterial capacities index (IMCI) (50). The third variable is the Political stability and absence of violence/terrorism, is one of the six world governance indicators created by the World Bank. PS measures the likelihood of violence and terrorism that can lead to government destabilization or being overthrown (51). The fourth variable is the Universal Health Coverage (UHC) service coverage index (52). A detailed description of the data and its sources is provided in Supplementary material. The selection of these four variables for analysis in this study is grounded in the existing literature, where scholars have identified these factors as crucial determinants of disparities in the distribution of COVID-19 vaccines among countries (18, 19, 21, 22, 26–32, 34).

Originally, 217 countries were included in the study. However, each of the previously mentioned variables had missing data points. Thus, the analysis in this study is limited to countries that had data for each variable, resulting in a final number of 163 countries being included. Given that complete data for all variables necessitated a reduction in sample size from 217 to 163 nations, the possible implications for the study’s findings and generalizability should be carefully considered. The refined sample adds a trade-off in terms of external validity, but it guarantees a more uniform dataset about the variables being studied.

## 3 Results

The analysis for TVH and VPP revealed significant disparities in COVID-19 vaccine distribution among countries. The CI analyses demonstrated inequalities in both TVH and VPP based on the four indicators. The MLR proved the presence of a significant relationship between COVID-19 vaccine distribution and PS, WPI, and UHC, but not GDP/capita. The breakdown of the results is detailed below.

### 3.1 TVH

**Figure 2:**
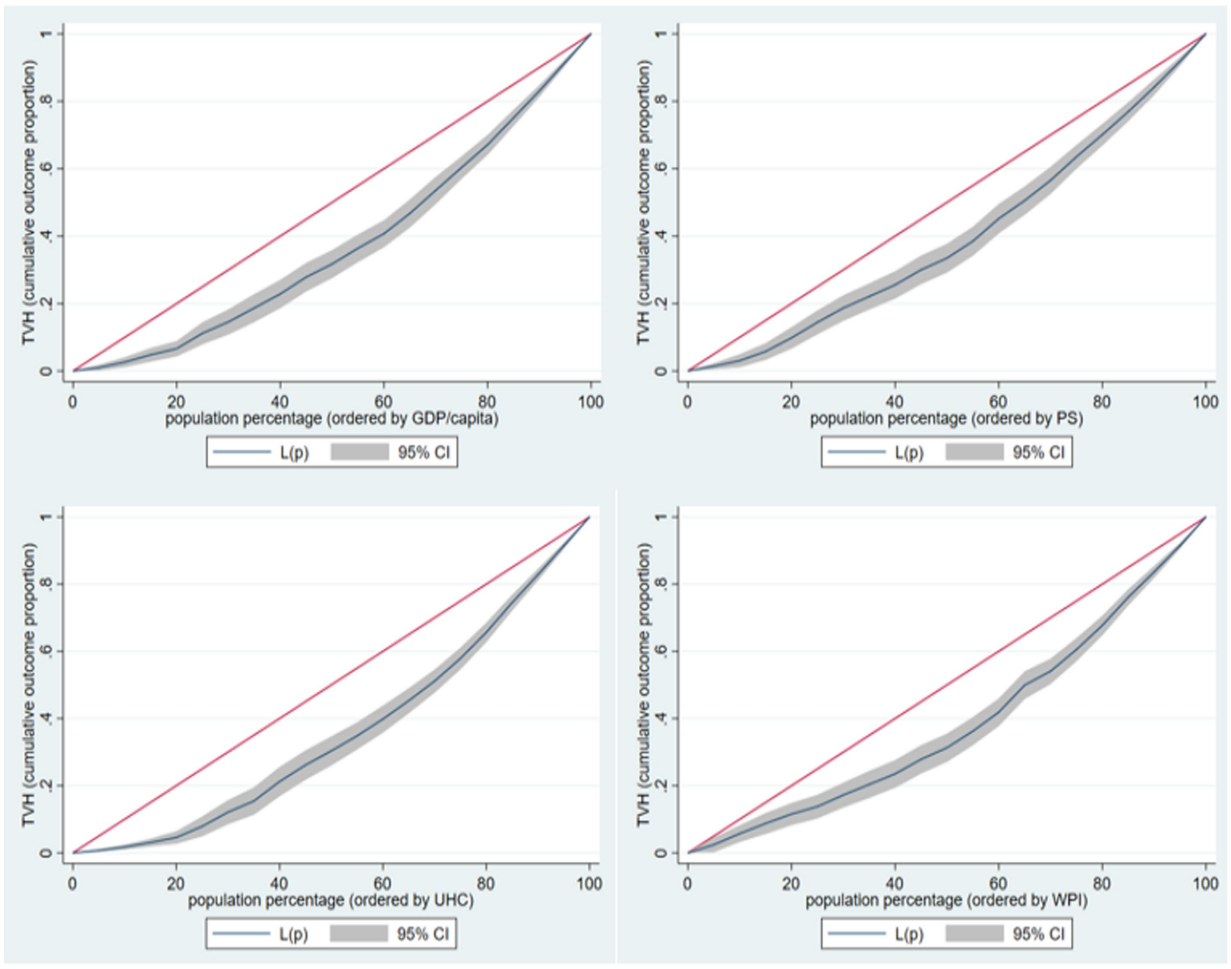
Concentration index analysis for TVH.

The CI analysis for TVH against the four indicators used as stratifiers (Figure 2) indicates the presence of inequality in TVH according to these countries’ GDP/capita, PS, WPI, and UHC.

The CI values for GDP/capita PS, WPI, and UHC range between 0.209 and 0.284, with the UHC having the higher CI value and PS having the lowest CI value (Table 1).

**Table 1:** Concentration index analysis results for TVH.

TVH		
	Concentration index	Standard error
GDP	0.25437368	0.02329784
PS	0.20912560	0.02389015
WPI	0.22627667	0.02349742
UHC	0.28401462	0.02313902

The MLR model for TVH against GDP/capita, PS, WPI, and UHC turned out to be statistically significant, with an *F*-value of 69.33 and a *p*-value of less than 0.001. The model explained 68% of the variability in TVH. Among the four variables: GDP/Capita was statistically insignificant, while PS, WPI, and UHC were statistically significant, indicating that these three are the ones related to TVH and can be used to explain the variation in its values (Table 2).

**Table 2:** TVH multiple linear regression model.

TVH			
	*β*	t	*p*
Constant		−6.119	<0.001
GDP/capita	−0.082	−1.124	0.263
WPI	0.199	2.266	0.025
PS	0.337	5.073	<0.001
UHC	0.477	5.780	<0.001
Model summary	R^2^ = 0.68, *F* = 69.33, *p* < 0.001		

### 3.2 VPP

**Figure 3:**
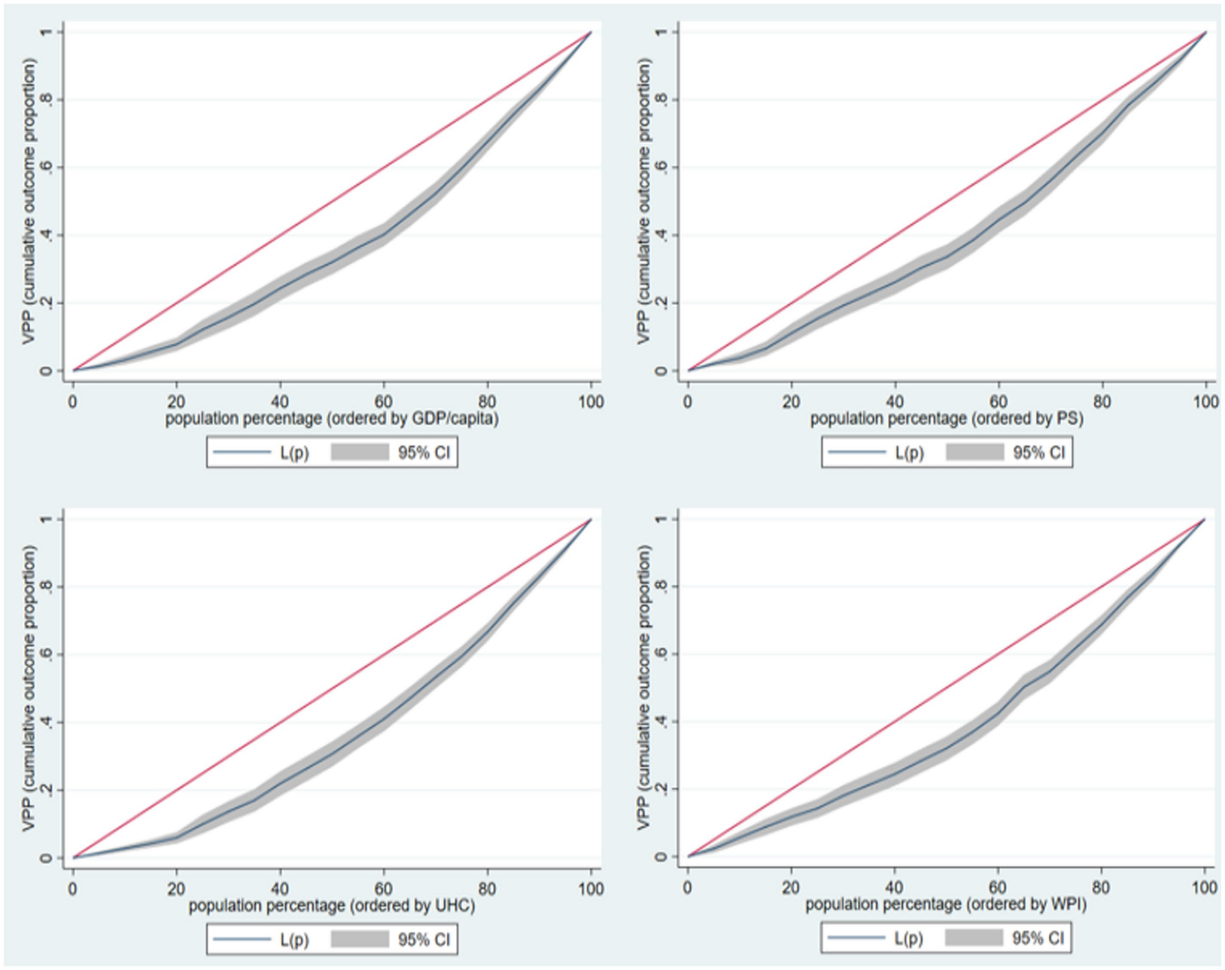
Concentration index analysis for VPP.

Similarly, to TVH, the CI analysis results for VPP against the four indicators used as stratifiers (Figure 3) indicate the presence of inequality in VPP according to these countries’ GDP/capita, PS, WPI, and UHC.

The CI values for PS, WPI, GDP/capita, and UHC range between 0.201 and 0.263, with the UHC having the higher CI value and PS having the lowest CI value (Table 3).

**Table 3:** Concentration index analysis results for VPP.

VPP		
	Concentration index	Standard error
GDP	0.24767798	0.01990379
PS	0.20061547	0.02087281
WPI	0.21636005	0.01920256
UHC	0.26347286	0.01996742

The MLR model for VPP against GDP/capita PS, WPI, and UHC turned out to be statistically significant, with an *F*-value of 70.746 and a *p*-value of less than 0.001. The model explained 69% of the variability in VPP. Among the four variables: GDP/Capita was statistically insignificant, while PS, WPI, and UHC were statistically significant, indicating that these three are the ones related to VPP and can be used to explain the variation in its values (Table 4).

**Table 4:** VPP multiple linear regression model.

VPP			
	*β*	t	*p*
Constant		−5.082	<0.001
GDP/capita	−0.015	−0.212	0.833
WPI	0.257	2.941	0.004
PS	0.338	5.126	<0.001
UHC	0.403	4.922	<0.001
Model summary	R^2^ = 0.69, *F* = 70.746, *p* < 0.001		

## 4 Discussion

The literature on the COVID-19 vaccine and its distribution among different countries has strongly highlighted the unequal access to the vaccine. Some relate this inequity to factors that pertain to the country itself, namely its human and technological capacities, while others discuss external factors such as power imbalances in the global health arena, global solidarity, and health security. Almost all of these studies are of qualitative nature or descriptive at the very least. To the best of our knowledge, there is limited or no existing research that specifically utilizes the combination of our chosen methods, namely CI analysis and MLR, in the context of global-level COVID-19 distribution analysis. This research measures the inequality in COVID-19 vaccine distribution according to countries’ economic status, political power, PS, and health system strength, followed by investigating the relation between the intercountry COVID-19 vaccine distribution and the four variables (i.e., economic status, political stability, political power, and health system strength) with the aim of determining which of these factors is most important to address in order to enhance the accessibility to vaccines in future pandemics, which will indirectly lead to better equality in distribution.

The CI analysis results for both VPP and TVH proved the presence of inequity in the distribution of COVID-19 vaccines among countries according to the countries’ GDP/capita, PS, WPI, and UHC. The sign and the magnitude of the concentration indices for both indicators indicate that countries with high GDP/capita, PS, WPI, and UHC had higher accessibility to vaccines. This finding is concerning since it implies that countries with lower GDP/capita, PS, WPI, and UHC are facing greater challenges in accessing and administering COVID-19 vaccines, which exacerbate global health inequities and prolong the pandemic by allowing the COVID-19 virus to circulate in countries with lower vaccination rates, leading to potential outbreaks and the emergence of new variants. GHG policies ought to pay significant attention to countries with lower GDP/capita, PS, WPI, and UHC. GHG ought to secure and satisfy the need of these less fortunate countries for vaccines through international collaborations. Efforts should focus on bridging the gap between high- and low-income countries by facilitating vaccine access, increasing production capacities, and providing financial and technical support to countries in need in order to achieve global health equity and global health security.

Although the CI analysis indicated the presence of inequality in COVID-19 vaccine distribution according to their GDP/capita, PS, WPI, and UHC, unexpectedly, the regression analysis showed that there is no significant relationship between COVID-19 vaccine distribution and the countries’ GDP/capita. This interesting result means that although economic-based inequalities are present, the economic status of countries is not a main factor in affecting COVID-19 vaccine distribution but other factors. GDP/capita can enable the country to purchase the vaccine; however, it is not the determinant of COVID-19 vaccine distribution among and within countries. Other external and internal factors are involved. The regression analysis proved that both TVH and VPP are significantly related to the three other factors: WPI, PS, and UHC.

UHC had the highest significant positive coefficient, suggesting that in countries with higher levels of UHC, the quantity of COVID-19 vaccine doses delivered was higher. UHC is an indicator used as a proxy for the strength of the health system in a country. A country providing a decent level of UHC needs to have a relatively strong health system that can provide services and cover the population. A strong health system would facilitate the management of the COVID-19 vaccines, from procurement to distribution and finally administration. A strong health system means having the financial and human resources to accomplish adequate COVID-19 vaccine coverage for their population. This justifies the strong relationship between UHC and both TVH and VPP.

The second most significant predictor variable is the PS in a country. PS reflects the country’s context in terms of its ability to prioritize COVID-19 as a primary concern, its ability to import or manufacture vaccines, raise people’s awareness enough to accept vaccines, and the ability of the health system to function efficiently to administer vaccines. These governments or responsible parties do not have the required financial resources to acquire vaccines, and health may not be their top priority due to the political and sovereignty issues they need to concentrate on. COVID-19 has exacerbated the existing health inequities in these countries as well as added new ones, such as the inequity in accessibility to the COVID-19 vaccine. The inequity in the intercountry distribution of COVID-19 vaccines is not solely determined by socioeconomic factors within these countries. It extends to the global level, where entire nations are discriminated against by the global community in terms of vaccine allocation, particularly in the absence of strong global solidarity.

The third predictor variable related to COVID-19 vaccine distribution is WPI. The power of a state can stem from several attributes: economy, politics, military, culture, technology, etc. These attributes enable the state to make decisions and execute measures that it deems necessary to preserve its sovereignty. The COVID-19 pandemic threatens states at multiple levels: population health, economy, PS, and even their sovereignty. In the presence of a power imbalance among states and the absence of a GHG structure with the power to lead all states to a unified response against the pandemic, these powerful states prioritized their populations and interests regardless of the consequences of their decisions on other nations. Powerful states were capable of securing COVID-19 vaccines for their populations. Some of these powerful states secured more than double their needs, while weak nations struggled to secure the minimum amount of vaccine to cover the most vulnerable segment of their populations.

Acting on the above-mentioned factors (UHC, PS, and WPI) can enhance accessibility to the COVID-19 vaccine in countries and ultimately enhance equity in COVID-19 vaccine distribution. According to the results of the MLR, UHC has the highest coefficient meaning that working on enhancing countries’ UHC achievement will have the highest return on improving the accessibility of vaccines in future pandemics. UHC is based on the notion of equity. The aim of UHC is to ensure everyone’s accessibility to essential health services, including vaccines, without facing financial hardship. UHC requires strong health systems with enhanced capacity to store, distribute, and administer vaccines effectively, as well as ensure that vulnerable populations and marginalized communities have equal access to COVID-19 vaccines. Strengthening health systems and progressing in the attainment of UHC should be a priority on local as well as global health agendas to enhance vaccine accessibility and equity.

Regarding PS, PS creates an environment conducive to long-term planning and investment in healthcare infrastructure. Governments can allocate resources to strengthen healthcare systems, including vaccination facilities, cold chain storage, transportation networks, and healthcare workforce training. A stable political environment encourages sustained investments in these critical areas, ensuring efficient vaccine distribution and accessibility. Moreover, PS ensures effective policy implementation and attracts international aid.

Finally, countries’ economic and social powers. During the COVID-19 pandemic, countries’ powers were exploited in ways that favored nationalistic gains instead of global gains. Equity and solidarity, and hence accessibility to COVID-19 vaccines, were not top priorities for countries. Nevertheless, countries’ power can be leveraged to increase accessibility to vaccines. Political power can be directed to influence global health policies, such as policies to reduce trade barriers and intellectual property restrictions that might hinder the production and distribution of vaccines. Economic power can be directed to finance vaccines, support their manufacturing, and increase production and supply. Finally, countries with knowledge power need to collaborate with other nations and share their scientific knowledge and expertise to accelerate vaccine development.

The findings of this study can have significant implications for both the global response to the COVID-19 pandemic and the existing literature on vaccine distribution disparities. The disparities in access to COVID-19 vaccines that have been identified and their underlying causes highlight the interconnected nature of global power structures, healthcare systems, and political dynamics. This addition will enhance the current discussion on vaccines and global health equity.

### 4.1 Study limitations

Although the study offers insightful information regarding COVID-19 vaccine distribution inequality among countries and the factors associated with this distribution, it has its limitations. The analysis focused on COVID-19 vaccine distribution inequality among countries based on specific factors such as economic status, PS, political power, and health system strength. While these factors are important, they are not the only ones affecting COVID-19 vaccine distribution. Nevertheless, these factors are supported by the literature and other peer-reviewed studies.

Another limitation is that the study used COVID-19 vaccine distribution data from a specific point in time (at the beginning of the vaccine distribution), which entails that the results of the study are valid for this period and might have changed in the period that came after. This does not reduce the importance of the results, as they demonstrate that in the period when vaccines were not produced in enough quantities, equality in distribution was ignored.

## 5 Conclusion

Inequalities according to countries’ GDP/capita, PS, WPI, and UHC are present. However, it appears that COVID-19 vaccine distribution is significantly related to PS, WPI, and UHC and not to GDP/capita. To improve the accessibility of vaccines in future pandemics, GHG and countries should consider working on three areas: enhancing PS in countries, separating political power from decision-making at the global level, and most importantly, supporting countries to achieve UHC. Regarding future research, more in-depth research is needed to identify the specific mechanisms through which PS, world power dynamics, and health coverage impact vaccine accessibility.

## Data availability statement

The original contributions presented in the study are included in the article/Supplementary material, further inquiries can be directed to the corresponding author.

## Author contributions

WA: Methodology, Data curation, Formal analysis, Investigation, Writing – original draft, Writing – review & editing. ZK: Formal analysis, Methodology, Writing – review & editing, Conceptualization, Validation. HE: Conceptualization, Validation, Supervision, Writing – review & editing. SC: Conceptualization, Supervision, Validation, Methodology, Writing – review & editing.
