# Supplementary material for: COVID-19 vaccine intercountry distribution inequality and its underlying factors: a combined concentration index analysis and multiple linear regression analysis
Source: Front Public Health. 2024 Mar 21;12:1348088. doi: 10.3389/fpubh.2024.1348088 (PMC10993910; doi:10.3389/fpubh.2024.1348088)
Supplement: Supplementary file 1 [file Data_Sheet_1.docx]

Supplementary materials

# Countries included in the study

| Burundi | Central African Republic | Afghanistan | Mozambique |
| --- | --- | --- | --- |
| Sierra Leone | Madagascar | Niger | Congo, Dem. Rep. |
| Malawi | Liberia | Chad | Guinea-Bissau |
| Gambia | Burkina Faso | Uganda | Rwanda |
| Ethiopia | Mali | Tajikistan | Togo |
| Guinea | Tanzania | Lesotho | Nepal |
| Benin | Pakistan | Zambia | Haiti |
| Zimbabwe | Syrian Arab Republic | Kyrgyz Republic | Comoros |
| Senegal | Cameroon | Timor-Leste | Cambodia |
| Uzbekistan | Bangladesh | Kenya | Nicaragua |
| India | Nigeria | Ghana | Cote d'Ivoire |
| Solomon Islands | Congo, Rep. | Honduras | Lao PDR |
| Vietnam | Angola | Papua New Guinea | Egypt, Arab Rep. |
| Iran, Islamic Rep. | Vanuatu | Morocco | Bhutan |
| Djibouti | Philippines | Bolivia | Tunisia |
| Cabo Verde | Ukraine | Sri Lanka | Eswatini |
| Algeria | Indonesia | El Salvador | Samoa |
| Mongolia | Jordan | Moldova | Armenia |
| Guatemala | Georgia | Azerbaijan | Tonga |
| Namibia | Belize | Jamaica | Paraguay |
| Albania | Iraq | Bosnia and Herzegovina | Fiji |
| Ecuador | Colombia | Guyana | South Africa |
| Belarus | Suriname | Peru | Botswana |
| Serbia | Lebanon | Turkmenistan | Libya |
| Gabon | Thailand | Dominican Republic | Equatorial Guinea |
| Brazil | Montenegro | Turkey | Cuba |
| Bulgaria | Mexico | Argentina | China |
| Grenada | Mauritius | Malaysia | Russian Federation |
| Costa Rica | Romania | Chile | Croatia |
| Poland | Panama | Trinidad and Tobago | Hungary |
| Antigua and Barbuda | Uruguay | Oman | Latvia |
| Barbados | Greece | Lithuania | Saudi Arabia |
| Portugal | Estonia | Bahrain | Czech Republic |
| Slovenia | Cyprus | Spain | Brunei Darussalam |
| Korea, Rep. | Kuwait | Italy | Bahamas, The |
| France | Japan | United Arab Emirates | New Zealand |
| United Kingdom | Israel | Canada | Belgium |
| Germany | Finland | Austria | Sweden |
| Netherlands | Australia | Denmark | Qatar |
| United States | Singapore | Iceland | Norway |
| Ireland | Switzerland | Luxembourg |  |

# Data sources and study variables

### Total vaccinations per hundred (TVH)

*TVH* is the number of vaccine doses administered per 100 people within a given population, including booster doses. All doses are counted individually. This indicator is obtained from Our World in Data website (1). Our World in Data is a project of the Global Change Data Lab based at the University of Oxford. The Covid-19 dataset for this website is obtained from official numbers provided by governments and health ministries around the world and is updated daily. As for population estimates for per-capita metrics they are based on the United Nations World Population data. The TVH data for this paper was extracted on 17/5/2022, it contains 203 data point with an average of 129.54 and ranges between 0.1 and 355.75.

### Vaccine courses delivered as a proportion of country population (VPP)

VPP is the number of vaccine doses (in a full course for a given vaccine) as a proportion of country population. This indicator data is obtained from the UNICEF COVID-19 Vaccine Market Dashboard (2). The website vaccine data is compiled from public sources and complemented with relevant information from Airfinity intelligence platform. Vaccine data does not consider countries’ vaccination strategies whether the country prioritize vaccine use for first dose or for full dose vaccination. As for population estimates, they are obtained from UN population division 2019 revision. Dashboard data is updated weekly. Data for this study was extracted on 25/2/2022. The number of data points available were 175 points with an average of 75.91 and ranges between 2.04 and 181.67.

### Gross Domestic product per capita (GDP)

“GDP per capita is gross domestic product divided by midyear population. GDP is the sum of gross value added by all resident producers in the economy plus any product taxes and minus any subsidies not included in the value of the products. It is calculated without making deductions for depreciation of fabricated assets or for depletion and degradation of natural resources” (3). GDP per capita data for this study is of the year 2019 and was obtained from the World Bank data website: (<https://data.worldbank.org/indicator/NY.GDP.PCAP.CD>) . Number of data points available was 205 points with an average of 18605.51 and ranges between 228.21 and 189487.15. From the data obtained from the World Bank website, a categorical variable was constructed with four categories (Low-Income Countries LICs, Lower Middle-Income Countries LMICs, Upper Middle-Income Countries UMICs, and High-Income Countries HICs). Categories were based on the World Bank by income country classification.

### World Power Index (WPI)

WPI is a composite index. It is composed of three subindexes that reflect the multi-dimensionality of the concept of state power. The three indexes are: Material capacities index (MCI), Semi-material capacities (SMCI), and Immaterial capacities index (IMCI). Each of the indexes is a composite index of a set of essential variables.

MCI represent the economic-military power of the state and is composed of six essential variables: national production (gross national income, Atlas method current US$), territorial area (Km2), defence (military expenditure, % of gross domestic product), international commerce (trade, % of gross domestic product), finance (total reserves including gold, current US$), and research and development expenditure (% of gross domestic product).

SMCI reflects the socio-institutional power of the state and is composed of six variables: production per capita (gross national income per capita, Atlas method current US$), Population (total), consumption (Household final consumption expenditure per capita US$), energy (Electric power consumption, kilowatt hour per capita), education (Spending on education, total as % of gross domestic product), and health (Health expenditure as % of gross domestic product)

IMCI measures the cultural-communicative power of the state and includes the following variables: government expenditure (General government final consumption expenditure, current US$), tourism appeal (International tourism, revenues, current US$), international aid (Net official development assistance (ODA) received per capita, current US$), media (Telephone lines), academic influence (Scientific and technical journal articles), and cosmopolitism (International migrant stock, total).

All the data used to calculate WPI is obtained from DataBank-World Development Indicators. WPI data can be accessed through the WPI index website (4). The latest data available is for the year 2017. The number of data points available were 176 points with an average of 0.465 and ranges between 0.136 and 0.954.

### Political Stability and Absence of Violence/Terrorism (PS)

PS is one of the six world governance indicators created by the World Bank. PS measures the likelihood of violence and terrorism that can lead to government destabilization or being overthrown. It is an aggregate indicator that gives countries scores. The scores range from approximately -2.5 to 2.5 (i.e., follow a standard normal distribution). This aggregate indicator is based on underlying data from different sources. Data used covers the following areas: Orderly transfers, Armed conflict, Violent demonstrations, Social unrest, International tensions / terrorist threats, Political terror scale, Security risk rating, Intensity of internal conflicts, Intensity of violent activities, Intensity of social conflicts, Government stability, Internal conflict, External conflict, Ethnic tensions, Protests and riots, terrorism, interstate war, civil war, Right to Freedom from Disappearance, Right to Freedom from Extrajudicial Execution, Right to Freedom from Arbitrary Political Arrest, Right to Freedom from Torture and Ill-Treatment, The risk of political instability is very low, Civil conflict is effectively limited^[[1]](#footnote-1)^. These data are rescaled and combined using a statistical methodology called unobserved components. PS data used for this study is of the year 2020 and is obtained from the World Bank Governance Indicators website (5). Number of data points available is 206 points with an average of 48.94 and ranges between 0 and 100.

### UHC Service Coverage Index 2019 (UHC)

UHC is an index that measures the essential health services average coverage. UHC measure the average coverage of 14 tracer indicators in four main areas: *first*, reproductive, maternal, newborn, and child health. *Second*, infectious diseases. *Third*, Non-communicable diseases. And finally, service capacity and access. The index is computed using geometric means of the 14 tracer indicators and reports on a unitless scale of 0 to 100. Data for the tracer indicators is taken from the most recent data from WHO or other international agencies. Data on UHC for this study is of the year 2019 and is obtained from the UHC Global Monitoring Report (6). Number of data points available is 192 points with an average of 64.53 and ranges between 27 and 89.

References

1. Ritchie H, Mathieu E, Rodés-Guirao L, Appel C, Giattino C, Ortiz-Ospina E, et al. Coronavirus (COVID-19) vaccination [Internet]. Our World in Data. 2020 [cited 2022 Jun 6]. Available from: https://ourworldindata.org/covid-vaccinations

2. UNICEF. COVID-19 Vaccine Market Dashboard [Internet]. UNICEF supply division. [cited 2022 Jun 6]. Available from: https://www.unicef.org/supply/covid-19-vaccine-market-dashboard

3. Glossary | DataBank [Internet]. [cited 2022 Jun 6]. Available from: https://databank.worldbank.org/metadataglossary/sustainable-development-goals-%28sdgs%29/series/NY.GDP.PCAP.KD

4. Morales Ruvalcaba, D. DatabaseWPI [Internet]. World Power Index. [cited 2022 Jun 6]. Available from: https://www.worldpowerindex.com/data-wpi/

5. WorldBank. WGI 2021 Interactive Data Access [Internet]. WorldBank Governance Indicators. [cited 2022 Jun 7]. Available from: http://info.worldbank.org/governance/wgi/Home/Reports

6. WHO, WorldBank. Tracking Universal Health Coverage: 2021 Global Monitoring Report. World Bank; 2021.

7. COVID-19 Vaccine Manufacturing [Internet]. Knowledge Portal. [cited 2022 Jun 7]. Available from: https://www.knowledgeportalia.org/covid19-vaccine-manufacturing

# Variance inflation factor analysis results

# Homoscedasticity (Breusch-Pagan Test) results


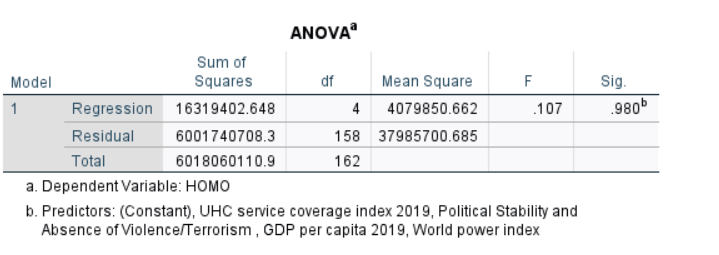


# Observation Independent (The Durbin-Watson test)


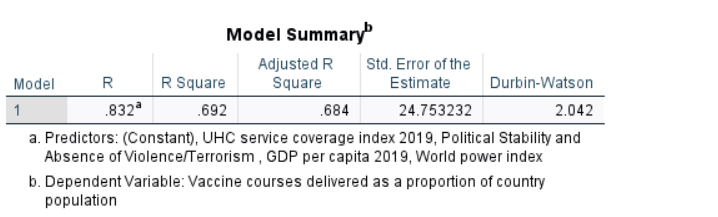


1. <file:///C:/Users/HD/Downloads/pv%20(1).pdf> [↑](#footnote-ref-1)
